# Application of tetraspanin CD81 RNAi for diagnosis and therapy of rheumatoid arthritis (RA)

**DOI:** 10.1186/ar3653

**Published:** 2012-02-09

**Authors:** Tohru Nakanishi, Yuji Arai, Hiroki Mori, Toshihiro Nakajima, Toshikazu Kubo

**Affiliations:** 1 Shujitsu University School of Pharmacy, Okayama, 703-8516 Japan; 2 Department of Orthopaedics, Kyoto Prefectural University of Medicine, Kyoto, 602-8566 Japan; 3 Institute of Medical Science, Tokyo Medical University, Shinjuku, 160-8402 Japan

## 

CD81 belomgs to a family of cell-surface protein (tetraspanin) which has four transmembrane domains and two outer-membrane loops.Under the DNA chip analysis, we found several genes highly expressed in rheumatoid arthritis (RA) synoviocytes comparing with the expression in OA or normal synoviocytes. Among these genes, tetraspanin CD81 was shown to be involved in the progression of RA through the promotion of Synoviolin expression.Synoviolin is already known as one of the important progressive elements of RA in synoviocytes. We also showed Synoviolin and CD81 highly distributed in RA tissues.

The therapeutic effect of small interfering RNA targeting CD81 (siCD81) was examined by *in vivo *electroporation method. Treatment with siCD81 significantly ameliorated paw swelling of collagen-induced arthritic (CIA) rats. In histological examination, hypertrophy of synovium, bone erosion, and degeneration of articular cartilage were minder in rats treated with siCD81 than in the control group and the non-specific siRNA group. Expression of synoviolin, a rheumatoid regulator, was also suppressed by siCD81 [1]. These results showed that siCD81 would become effective tools for treatment of RA. In addition, siCD81 reduced the amount of CD81 in synovial fluid indicating that quantitative analysis of CD81 opens up the novel and highly sensitive diagnosis for RA.

**Figure 1:**
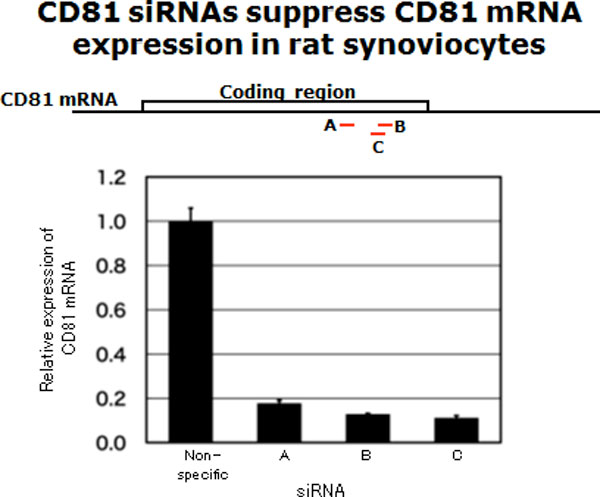


## References

[B1] Nakagawa Shuji Arai Yuji Mori Hiroki Matsushita Yumi Kubo Toshikazu Nakanishi Tohru Small interfering RNA targeting CD81 ameliorated arthritis in rats Biochem Biophys Res Commun 2009 388 467 472 10.1016/j.bbrc.2009.06.150 19580788

